# Risk Communication Distributed among Migrant Workers during the COVID-19 Crisis in Thailand: Analysis on Structural and Networking Gaps

**DOI:** 10.3390/tropicalmed7100296

**Published:** 2022-10-12

**Authors:** Hathairat Kosiyaporn, Sataporn Julchoo, Ratchadaporn Papwijitsil, Sonvanee Uansri, Mathudara Phaiyarom, Pigunkaew Sinam, Rapeepong Suphanchaimat

**Affiliations:** 1 International Health Policy Program, Ministry of Public Health, Nonthaburi 11000, Thailand; 2 Division of Epidemiology, Department of Disease Control, Ministry of Public Health, Nonthaburi 11000, Thailand

**Keywords:** public health emergency, risk communication, social network analysis, migrant worker, Thailand

## Abstract

Language and cultural barriers among migrant workers hamper access to health risk information. This study aims to explore health risk communication structure and processes and identify the communication network of migrant workers during the COVID-19 pandemic in Thailand. This study used a parallel mixed-methods design combined with in-depth interviews and questionnaires for social network analysis from November 2020 to June 2021 in the headquarter district of Samut Sakhon, Ranong, and Phuket provinces. We conducted purposive sampling of representatives from public and non-public organisations and local communities. Thirty-six key informants participated in in-depth interviews, and fifty-six respondents completed the questionnaire for social network analysis. Although health risk communication included various activities, there was no formal governing body responsible for health risk communication among migrants, and monitoring and evaluation of communication process were not well-implemented. The health risk communication network was centralised, especially in the rural area; however, migrant health volunteers (MHVs) and local media were key sources of information for most migrants in communities. Overall, a governing body led by the government with intersectional collaboration and a health risk communication process should be promoted while considering migrants’ characteristics and concerns. The health risk communication network should identify key communicators such as MHVs and local media. This can be an effective strategy to fill the gap of information dependency.

## 1. Introduction

Migrant workers face many challenges in accessing healthcare during the COVID-19 pandemic. This is due to several factors, including language differences, cultural barriers, financial hardship, and the precarious legal status of migrants [1]. In late December 2020, Thailand faced a new wave of COVID-19 which was believed to have originated in the migrant worker population [2]. This created a policy discourse on whether, and to what extent, migrant workers were able to access health-risk information to better prepare them and their families. Migrant workers in the informal sector, such as domestic workers or agricultural workers, tend to be missed from social protection or public insurance, leading to limited access to COVID-19 information [3]. In addition, migrants in factory settings or construction sites mostly live in crowded conditions that are prone to disease transmission [3]. It has been particularly important to discuss health risk communication in the dynamics of knowledge on disease transmission and preventive measures during the COVID-19 situation [4].

Public health emergencies highlight the importance of health risk communication, which aims to inform people to be aware of their risks and make decisions to protect themselves and their loved ones [5]. The health risk communication process includes monitoring people’s concerns and perceptions, assessing risks, developing communication activities, and evaluating impact [6,7]. To support this process, it is necessary for the government and partners to provide resources, alongside coordination of stakeholders, as recommended by World Health Organization (WHO) [8].

Health risk communication is recognised as important by the Emergency Operation Centre (EOC) in Thailand [9], and health risk communication planning for migrants is addressed by the Bureau of Risk Communication and Health Behavior Development, Ministry of Public Health [6]. However, poor coordination in previous emerging diseases has led to delay and inaccurate information for migrants [10,11]. The announcement of an emergency decree by the Center for COVID-19 Situation Administration also reflects centralised communication during the COVID-19 situation [12]. To ensure effective health risk communication for everyone, including migrants, resources and plans for migrants should be integrated into the overall emergency preparedness response.

Various health risk communication activities exist for migrant workers at both national and local levels in Thailand. The Sender–Message–Channel–Receiver (SMCR) Model by Berlo serves as a useful tool to evaluate the communication process as it includes senders, messages, channels, and receivers [13]. Senders are defined sources or people from where messages originate, while receivers are those who obtain the messages, try to understand what the sender actually wants to convey, and then respond accordingly (decoding) [14]. The message is the transformation of thoughts into words (encoding), and channel refers to the medium used to send the message such as broadcasting media or the Internet [14]. When applying the SMCR model in communication activities for migrant workers in Thailand, public organisations, including health facilities, non-governmental organisations (NGOs), employers, village health volunteers (VHVs), migrant health workers (MHWs), and migrant health volunteers (MHVs), play a sending role at the local level, while receivers are migrant workers and their family members in migrant communities [15,16]. A study by Mallinga et al. (2020) showed that the most common accessible message in Thailand among migrant workers is about preventive practices, whereas information about access to healthcare and public measures is scarce [17]. Additionally, information channels for migrant workers are also communicated through media such as television, helplines, documents, and online media [12,16]. Although there are studies about health risk communication activities for migrant workers in Thailand, the information network between sender and receiver has not been previously evaluated, and its challenges and recommendations not systematically discussed.

Research has not yet thoroughly explored health risk communication among migrants in Thailand during the COVID-19 pandemic, particularly from the angle of structure, process, and communication networks. Although one study on COVID-19 communication with migrants in Thailand exists, it was a quantitative study focused on individual knowledge, attitudes, and practices of migrants towards COVID-19 and descriptive analysis of communication channels [17,18]. This study, therefore, aims to explore the health risk communication structure and process at national and local levels, as well as to identify the communication network of migrant workers during the COVID-19 pandemic in Thailand in order to improve health risk communication responding to future emerging diseases.

## 2. Materials and Methods

**Figure 1:**
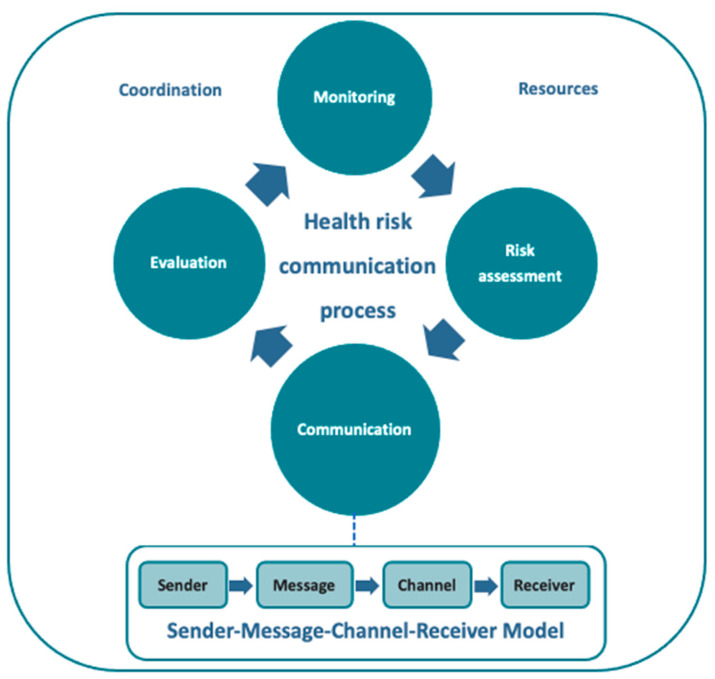
Conceptual framework.

The analysis followed the Concept of Risk Communication Process proposed by Bartram, Lang, and Fewtrell (2001) [7] in conjunction with the SMCR model by Berlo (1960) (see Figure 1) [13]. The process started from monitoring migrants’ concerns and perceptions, then assessing risks to guide communication activities and evaluation. The communication activities applied the SMCR model as a specific framework. Moreover, the overall framework is supported by coordination among stakeholders and resources [8].

### 2.1. Study Design

We used a parallel mixed-methods design of in-depth interviews and a survey in social network analysis in order to understand the health risk communication structure and process at national and local levels and identify the communication network between sources of information and migrant workers during the COVID-19 pandemic in Thailand. It was conducted from November 2020 to June 2021 in the headquarter district of Samut Sakhon, Ranong, and Phuket, which include some of the most densely migrant-populated provinces in Thailand [19]. We chose Samut Sakhon as it represents the central or urban area where most migrants are factory workers and construction labourers. Ranong and Phuket represent the border or rural areas; most of the migrant workers in Ranong work in the fisheries, while those in Phuket are service labourers. For social network analysis, we planned to conduct fieldwork in two migrant communities (jointly selected by the research team and local NGOs) per province. However, Phuket was excluded due to unreadiness for network analysis. After we conducted an initial assessment of communication channels among migrant workers, we found that there was a similar picture of migrant communities in Ranong, so we selected only one community from Ranong (Chumthong) and two communities from Samut Sakhon (Wat Noi Nang Hong and Ban Auea Arthorn Tha Chin).

### 2.2. Participants and Data Collection

Data collection techniques consisted of in-depth interviews and questionnaires. For in-depth interviews, we interviewed 36 key informants (KIs). The KIs were purposively selected from the authorities that played a critical role in migrant communication at national and local levels. The included organisations were based on the brief review of health risk communication networks among migrants during COVID-19 from news, proceedings, reports, and academic papers from an online search engine. Additional interviewees were selected on the basis of snowball sampling until the data were saturated. The KIs included central and local public health authorities (Department of Disease Control, Department of Health Economics and Health Security—migrant health insurance, Primary Health Care Division—training MHWs and MHVs, MOPH), international organisations (WHO Thailand and International Organization for Migration (IOM)), NGOs (World Vision Foundation and Raks Thai Foundation), academia (Public Health and Communication Arts Faculty at Mahidol University and Chulalongkorn University), MHWs (migrants who were hired as interpreters at public health facilities), and MHVs (migrants who volunteered to work with the public health facilities as intermediaries with migrant communities) in selected areas.

For the questionnaire, we purposively surveyed 56 respondents who were likely to be part of the communication network of the migrant community during the outbreak. These respondents encompassed a vast range of people responsible for migrant health or those who live in migrant communities; for instance, representatives of the public health authorities such as Provincial Public Health Office (PPHO), District Public Health Office (DPHO), main public hospitals and health centres, labour authorities such as the Provincial Labour Office (PLO) and Fishery Association (FA), local government (LG), NGOs, employers, community leaders, accommodation owners, village health volunteers (VHVs), MHWs, MHVs, and migrant workers themselves. The response from the questionnaire was used as input for social network analysis. 

Before starting the interview, all participants were informed about the study and were asked for consent to participate. Each interview took about 30–60 min. The in-depth interviews were conducted face-to-face by the research team, except with some interviewees who preferred online interviews. The interviews were audio-recorded and transcribed verbatim. For the questionnaire, we asked trained MHWs and local hospital staff to help collect data. A briefing between the research team and the questionnaire collector was conducted once before initiating the questionnaire.

### 2.3. Interview Topics and Questionnaire Design

The interview guide covered the topics (objectives, channels, and messages of health risk communication) related to previous and current health risk communication activities for emerging diseases including COVID-19. It also covered the issues of coordination with other organisations, supporting resources, monitoring and self-evaluation of communication methods, and challenges and recommendations for future health risk communication for migrant workers in Thailand. The questionnaire design for social network analysis was adapted from the name generator and interpreter questionnaire [20]. We asked respondents to describe which people and organisations they received messages from. In addition, we asked the respondents to rate the frequency of message receipt (1 = never, 2 = once a month or less, 3 = once a week or less, 4 = more than once a week, and 5 = every day).

### 2.4. Data Analysis

We analysed the interview data with deductive coding. Transcription data were cross-checked with personal field notes for accuracy and consistency. Data were triangulated by document reviews from organisations, for example, organisational action plans, a summary of calls from the migrant helpline, and an organisation website and Facebook page to ensure data accuracy. S.T. generated themes aligned with the conceptual framework, and S.T., H.K., R.P., and P.S. coded the transcription data into each theme. S.T. rechecked the final outcomes, and any disagreement was discussed until there was consensus among researchers. Interview data were coded into four themes: (i) health risk communication structure and process; (ii) health risk communication actors and networks; (iii) health risk communication activities; and (iv) challenges and recommendations of health risk communication. In the second theme, social network communication was analysed in matrices and then depicted by sociograms that presented the number of nodes, vectors, and key parameters such as distance and centrality between nodes. Nodes (circles) represented sources of information, while a vector (lines) represented the flow of information from which arrowheads pointed out from a sender to a receiver. Distance referred to an average shortest path from each node to each migrant worker, which means those who have the shortest communication distance to migrant workers [21]. The betweenness-centrality was calculated from the proportion of shortest paths that go through one node, determining the centralised/dependent communication of each actor in the network, whereas closeness-centrality was the number of nodes divided by the sum of the length of the shortest paths between the node and all other nodes so the low value was the longer distance to each node [21].

### 2.5. Ethics Consideration

This study received ethics approval from the Institute for the Development of Human Research Protections, Thailand (IHRP 985/2563). The participants had to read an information sheet or be verbally informed by interviewers about research objectives, methods, privacy consideration, and benefits and potential risks from this study. If they decided to join, they would consent in a written document and would receive USD 10 allowance after finishing the interview or survey.

## 3. Results

Thirty-six KIs were recruited for the interview. The participants consisted of central MOPH (*n* = 5), international organisations (*n* = 7), local MOPH (*n* = 10), NGOs (*n* = 5), academia (*n* = 3), MHWs (*n* = 3), and MHVs (*n* = 3) (see Table 1). There were 22 female and 14 male interviewees. For social network analysis, 56 KIs responded to the questionnaire survey, comprising 26 participants in Ranong and 30 participants in Samut Sakhon. They were local MOPH (*n* = 8), NGOs (*n* = 2), labour sector (*n* = 2), LG (*n* = 2), employers (*n* = 3), community leaders (*n* = 2), accommodation owners (*n* = 2), VHVs (n=3), MHWs (*n* = 4), MHVs (*n* = 3), and migrant workers (*n* = 25) (see Table 2). The number of male and female participants was nearly equal. The mean age of Ranong participants was 42.4, and the mean age of Samut Sakhon participants was 38.1. Most of the migrant workers in Ranong worked in the fishery sector, whereas those in Samut Sakhon mostly worked in the fishery industry and construction sector, and some were unemployed.

The four themes identified from the interviews and the sociograms are described below.

Theme 1: Lack of implementation and effective national body for health risk communication structure and lack of monitoring and evaluation of the health risk communication process for migrants.

Several actors were involved in health risk communication of public health emergency especially organisations relating to migrant health and disease control. Some were responsible for migrant health before this pandemic, for example, the Primary Health Care Division and Division of Health Economics and Health Security (MOPH), World Vision Foundation, Raks Thai Foundation, WHO, and IOM. In a public health emergency, the Department of Disease Control (DDC), MOPH, who is responsible for health risk communication, cover both Thai people and migrants in their plan as a part of the EOC. The Bureau of Risk Communication and Health Behavior Development, DDC, said they played role in national health risk communication in terms of communication planning, monitoring media and public perceptions, risk assessment, coordination with organisations both within and outside the country, and supporting capacity building. However, the communication strategy for migrants addressed by the Bureau of Risk Communication and Health Behavior Development was not mentioned by stakeholders. Some KIs noted there was still no focal point in migrant health, which was supposed to cover health risk communication in emergency preparedness. Nevertheless, another KI noted that health risk communication work at the central level was well constructed but that there was an unclear structure at local level. For example, some provinces were assigned to a health communication subdivision while others were assigned to a disease control subdivision or migrant-related subdivision.

*“It seems like Thailand is a champion in migrant health. The strength is MOPH and partners have been working on this issue for a long time. However, the weakness is no focal point or sustainable platform”.*

(D4)

*“The scope and structure of health risk communication at central level (MOPH) is clear, but when it is transferred to local level, it is unclear”.*

(B1)

Overall health risk communication for Thai populations was conducted through various forms, such as mass media, online platforms, helplines, and a survey of public perceptions. Nevertheless, the national authorities (DDC, MOPH), coordinating with partners, usually promoted mass communication for migrant workers through the helplines (with bilingual interpretation) and online media. Local actors (such as MHWs, MHVs, and employers of migrant workers) mostly communicated with migrant members via personal connection and local media using printed documents and online media arranged by the local authorities (such as the PPHO, DPHO, health facilities, and NGOs); all were adjusted to the contexts. There were few communication activities provided specifically for migrant workers, and the monitoring and evaluation (M&E) of the communication was limited only to Thai people. The DDC conducted a series of surveys to monitor people’s concerns, perceptions, and preventive practices, but this, however, was conducted in the Thai language. Although the data from the migrant helpline were analysed to identify key concerns among migrants, the helpline was not well enough promoted to reach local migrant communities.

*“Interviewer: Are there any polls (to monitor people’s concerns and perceptions) provide in foreign language?*

*Interviewee: No, it is provided for those who can read Thai. When we got information from migrants, we send it to helpline 1422. Therefore, only migrants who can read Thai are able to participate”.*

(A2)

*“There was also a misconception of health risk communication work among staff. Some of them thought it was only about public relations (PR) or communication campaigns rather than a process starting with monitoring the situation and people’s perceptions, followed by implementation and evaluation”.*

(A2)

Financial and human resource support was also lacking. This situation created many unintended consequences; for example, limited budget hampered the hiring of additional interpreters to meet the demand and the production of bilingual infographics. A key source of funds to control communicable diseases among migrant workers is from the Health Insurance Card Scheme; the migrant insurance organised by MOPH was limited so other stakeholders had to allocate their budget to support. Furthermore, health risk communication officers were less promoted by policymakers than other health officers, and capacity building was not encouraged.

*“We do not have enough budget. But it is necessary to produce bilingual infographics. Accordingly, we used our limited budget to print A3 posters, then we disseminated them to other organisations”.*

(E1)

*“For capacity building, we suggest developing capacity about health risk communication in all organisations. We are not sure that we are going in the right direction or how to engage people… PPHO should have a team or person who are consultants for other organisations in province”.*

(B2)

Theme 2: Centralised health risk communication networks in migrant communities.

Various actors in health risk communication at local level were message-senders or sources of information. In Samut Sakhon, communication in the migrant communities was more accessible and more frequent than in Ranong. Blue nodes referred to authorities; orange nodes referred to migrant workers; and green nodes referred to media such as broadcasting media (television/radio), printed media (posters/leaflets), and online media at national and local levels (see details in the Supplementary File, Tables S1–S3). The thickness of edge determined frequency of communication, ranging from never (score 1), once a month or lesser (score 2), once a week or lesser (score 3), more than once a week (score 4), to every day (score 5).

**Figure 2:**
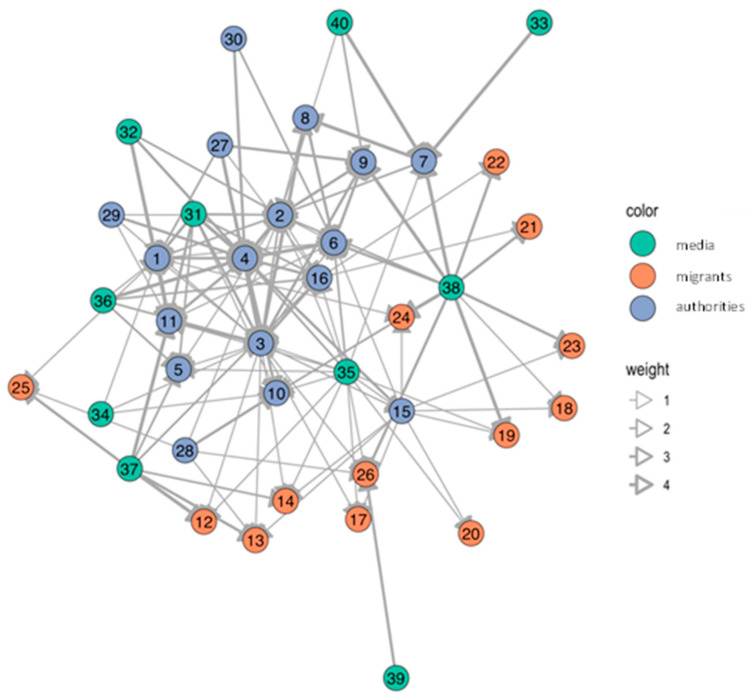
Network of communication in the Chumthong migrant community, Ranong.

**Figure 3:**
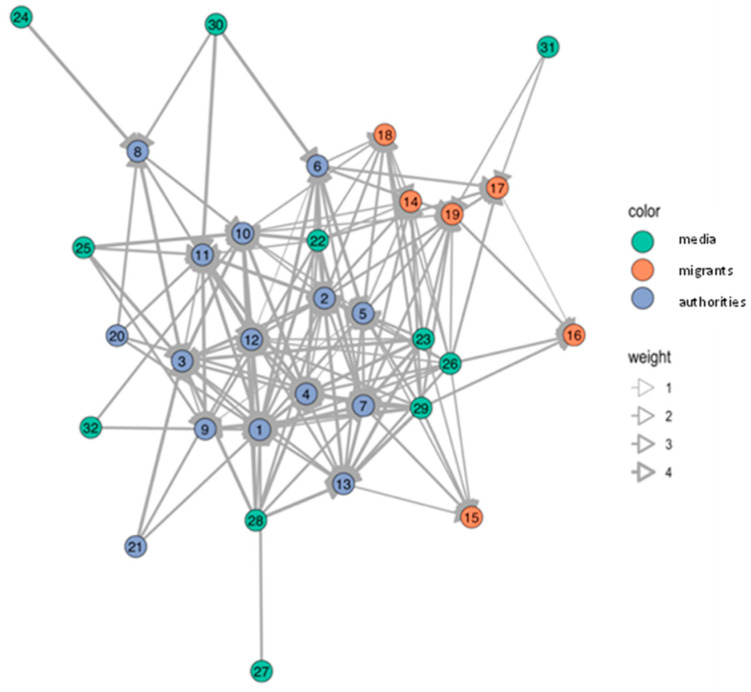
Network of communication in the Wat Noi Nang Hong migrant community, Samut Sakhon.

**Figure 4:**
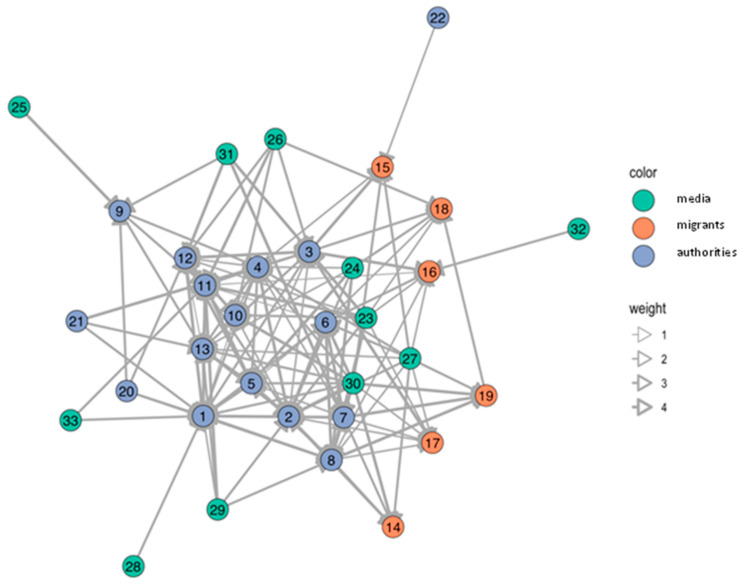
Network of communication in the Ban Auea Arthorn Tha Chin migrant community, Samut Sakhon.

The number of network nodes in Ranong was 40, and in Samut Sakhon, there were 32 and 33 in Wat Noi Nang Hong and Ban Auea Arthorn Tha Chin, respectively (see Figure 2, Figure 3 and Figure 4). Although the number of nodes in Ranong was higher than Samut Sakhon, the vectors in the two communities in Samut Sakhon (*n* = 158 in Ban Auea Arthorn Tha Chin and *n* = 144 in Wat Noi Nang Hong) were more populated compared with the community in Ranong (*n* = 130). The overall mean distance across nodes in Ranong was 2.5, while in Samut Sakhon was 1.9. It meant that the flow of information in Samut Sakhon was easier to access by all nodes, compared with Ranong.

**Figure 5:**
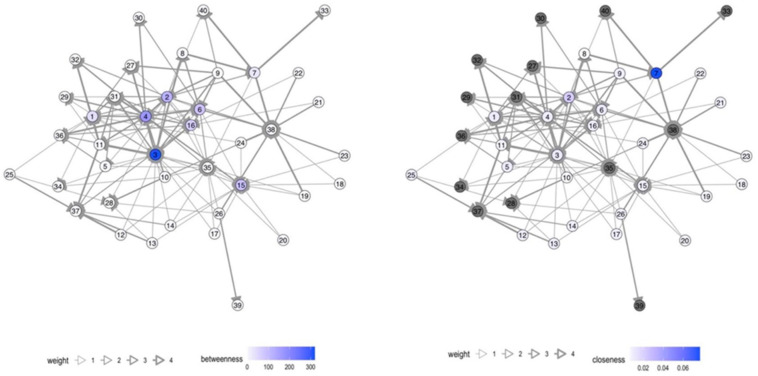
Betweenness- and closeness-centrality in the Chumthong migrant community, Ranong. Note: grey nodes refer to the nodes that are not applicable to calculate.

**Figure 6:**
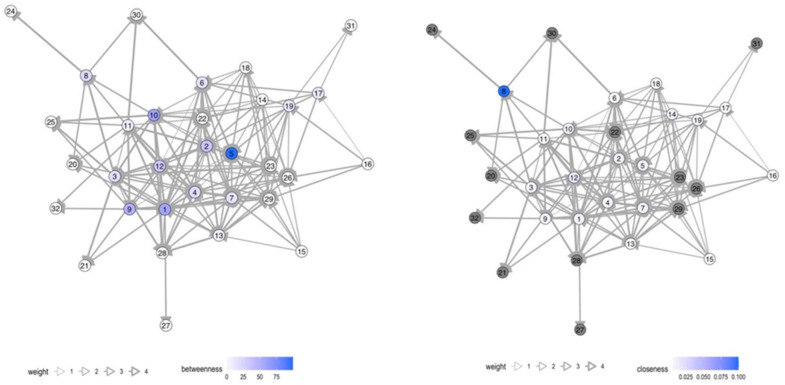
Betweenness- and closeness-centrality in the Wat Noi Nang Hong migrant community, Samut Sakhon. Note: grey colour refers to the nodes that are not applicable to calculate.

**Figure 7:**
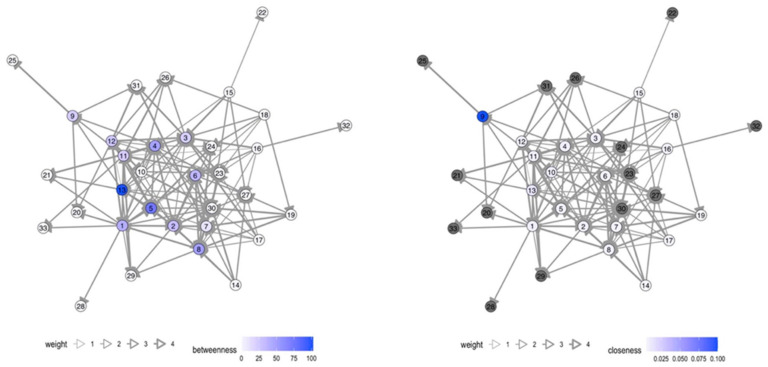
Betweenness- and closeness-centrality in the Ban Auea Arthorn Tha Chin migrant community, Samut Sakhon. Note: grey colour refers to the nodes that are not applicable to calculate.

Ranong saw a higher value of betweenness-centrality compared with Samut Sakhon. PPHO in Ranong (node 3) showed the highest betweenness-centrality (score = 319.7), followed by MHVs (node 4 score = 173.3) and DPHO (node 2 score = 119.8) (see Figure 5). Migrant workers mostly had the low closeness-centrality score less than 0.01. On the other hand, in Samut Sakhon, the value of betweenness-centrality had a far lower score (see Figure 6 and Figure 7). The highest betweenness centrality in Samut Sakhon was found in MHVs (node 5 score = 99.5), PPHO (node 1 score = 58.4), and health-promoting hospital (HPH) (node 10 score = 59.2) in Wat Noi Nang Hong, and local government (node 13 score = 102.4) and MHVs (node 5 score = 75.8) in Ban Auea Arthorn Tha Chin. Migrant workers in Wat Noi Nang Hong and Ban Auea Arthorn Tha Chin also had the lowest closeness-centrality score, accounting for 0.006 and 0.005, respectively. It means the communication was centralised in some senders or depended on those who had high betweenness-centrality, especially in rural areas, and migrant workers found it hardest to access information, representing a low closeness-centrality score. Please see the Supplementary Materials for more details (Tables S1–S3).

The average shortest communication distance to migrant worker nodes for Ranong was found in PPHO (node 3 score = 1.6), MHV (node 15 score = 1.6), local printed media (node 35 score = 1.6), and online media (node 38 score = 1.9). For the communication network in Samut Sakhon, the shortest communication distance to migrant workers was observed in local printed media (node 26 score = 1.0), local online media (node 29 score = 1.2), and MHVs (node 5 score = 1.3) in Wat Noi Nang Hong, and local printed media (node 27 score = 1.2), local online media (node 30 score = 1.3), employers/colleagues (node 3 score = 1.3), and hospital staff (node 8 score = 1.3) in Ban Auea Arthorn Tha Chin (see the Supplementary Materials, Tables S1–S3). It means the most common sources of information that migrant workers easily accessed were MHVs, printed media, and online local media in all areas.

Theme 3: Existence of diverse channels in health risk communication, but the accessibility, understanding, relevance, and timeliness were still a key concern.

The objectives of health risk communication were to inform people about preventive practices and public measures, including reducing fear, worry, and stigmatisation of infected groups. From the sender side, interviewees said that the most common communication methods about prior emerging health threats (such as SARS, H1N1 influenza, and MERS) were the distribution of paper-based documents (such as posters and leaflets) and through MHVs. During the COVID-19 pandemic, there was a combination of traditional and innovative media, such as the use of online infographics and videos. There was also additional training for MHVs and attempts to include migrant workers, employers of migrants, and owners of migrant accommodation to participate in increasing health risk communication.

*“Health risk communication is important because they (migrants) are fearful (of disease). The communication will help them understand and relieve their fears. They will know that it is not easy to get infected…if they have knowledge or exchange any information, they will be less worried”.*

(C2)

Regarding communication channels, the migrant helpline and bilingual infographics were initiated at the national level. The helpline for Cambodian, Laotian, and Burmese languages was operated by the DDC with volunteer interpreters from NGOs and international organisations. The helpline operators were tasked to respond to basic questions about disease and preventive and social measures, under the supervision of staff. Operators were also tasked to inform the disease investigators of any suspected or infected cases. At the end of the day, volunteer operators had to report a number of line calls and types of questions into the system. KIs said that it was helpful to make migrant workers more comfortable when they talked with people who have the same languages or cultures.

*“The migrant helpline is a good method because it is two-way communication. It can go into details of issues that migrants are concerned about. Not only talking about knowledge, this channel can also help migrants feel comfortable when they talk with those from the same countries”.*

(A1)

The bilingual infographics were translated and designed with coordination between the MOPH, WHO, Thai Red Cross Society, Thai Health Foundation, Raks Thai Foundation, World Vision Foundation, Labour Protection Network, Mahidol University, and others on the basis of the resources and expertise of each organisation. The video and animation were also popular, especially for those who are illiterate. These materials were disseminated through public organisations or NGO websites and adapted by local organisations in different contexts.

*“If we applied bilingual material from academia, it is usually in formal language and the migrants might not understand. Thus, we (NGOs) revise it to language used in migrant communities”.*

(C1)

During this period, MHVs were increasingly recruited in communities, workplaces, and field hospitals with support from the central government, employers, and health workers. In addition, the MOPH initiated a 10 h COVID-19 training course for MHVs. Those completing the course would acquire a certificate as proof of attendance. Furthermore, Samut Sakhon PPHO and Samut Sakhon hospitals initiated a training programme for MHVs in the field hospitals. The difference from the previous MOPH’s training was it also taught MHVs some basic clinical skills, such as vital-sign monitoring at the field hospital and assisting health personnel to facilitate the quarantine of people identified as infectious.

*“The MHVs are part of health services supporting health staff to take care of COVID-19 patients. Firstly, we recruit those who can speak Thai and have a volunteerism mindset, then we train them for a few hours. They monitor blood pressure and oxygen levels of other patients every day throughout the stay in the field hospitals”.*

(B7)

Some KIs mentioned that social media was an important communication channel as migrant workers could easily access it directly, and many forms of media, such as infographics or videos, were attractive. These respondents considered paper-based media as ineffective, arguing that online media was more appropriate in this era as most migrant workers were able to access smartphones and the Internet. Some migrants communicated with their friends and families (both inside and outside the country) through Facebook Messenger. Therefore, migrant-related organisations communicated with migrant workers by creating Facebook pages, such as “Migrant Health Network in Thailand” (created by a group of NGOs at the national level), “Migrant Health Volunteers” (created by Samut Sakhon Hospital), or “Ranong Thadin” (created by a group of NGOs in Ranong). Another communication channel was a public address system in the community, which was a station or a mobile system run by community leaders or public health organisations. The system sent health risk messages directly to migrant workers on a weekly basis.

*“Printed media is wasteful. It is easy to count the number of paper that we print or disseminate but we do not know if it is working or not”.*

(D5)

*“We make a video about quarantine and share in Facebook group consisted of NGOs who work with Burmese migrants…after they see the link and then share, the engagement is around 1000 to 10,000 views, thus, Facebook is the best channel at this moment”.*

(C4)

Health risk communication messages are mostly focused on knowledge about disease and preventive practices. Nevertheless, feedback from the migrant helpline showed that most concerns were about updated situations and measures including public health, labour, and border control policies. Queries about disease screening and quarantine measures were also common among migrant workers.

*“Most migrant workers ask about risks for themselves and close persons and the situation in the country. Sometimes, they refer to the situation in their own country and also ask about updated public measures in Thailand such as quarantine measures”.*

(D7)

On the receiver side, some migrant workers could not access online media, owing to lack of smartphones, limited access to the Internet, and poor digital literacy. Some lived in areas that authorities could not reach or were overlooked, such as domestic workplaces or in the Thai community. Furthermore, although the majority of migrant workers were from Myanmar, there were many more ethnic subgroups and groups of people with different languages used (for example, Karen and Mon). This diversity created a huge challenge for communication, as bilingual translation might not suffice in some settings.

*“Some Burmese migrant workers cannot use Facebook. Some do not have smartphones … especially the elderly, they cannot use it”.*

(F3)

*“Not all migrant workers live in the construction camps, some might work in small businesses or live in rental rooms in the Thai community. Thus, we cannot reach them due to the scattered accommodation, and they are left behind”.*

(B2)

Theme 4: The way forward for health risk communication for migrants in Thailand.

Improvements are required in health risk communication for migrants. First, the government should be a lead actor of a migrant information hub, facilitating collaboration among partners. KIs suggested it was necessary to recruit stakeholders from outside the health sector and work together in terms of information and resource sharing. For example, the Ministry of Labour (MOL) should collaborate with the MOPH to encourage the private sector to communicate public health measures (an MOL responsibility).

*“The government should be a centre of health risk communication…they should provide information and financial support, which will be a long-term development (of the health risk communication for migrants)”.*

(D6)

Second, the government should provide adequate resources and expertise for maintaining and improving health communication channels for migrants. This includes regular monitoring for reactive and proactive issue management and evaluation of the communication methods.

*“Issue management is management of information. We have to monitor social trends, and then define communication issues. Therefore, it will be useful for overall health risk communication in order to dealing with the chaos and panic resulting from misinformation… there can be parallel work of reactive issue management, which is a monitoring of public sentiments, and proactive reactive issue management, which is a forecast of future social trends”.*

(E3)

Third, the information disseminated to migrants should be accessible, understandable, and timely on the basis of different ethnicities, languages, cultures, and literacy skills. Common communication channels and information for migrants should be tailored.

*“Communication management should be tailor-made. It is not only one model for all areas, but has core principles. The details are supposed to be adapted based on environment and contexts”.*

(E3)

Finally, some KIs addressed the importance of MHVs in this situation, which was beneficial not only for health risk communication but for overall migrant health. They suggested improving migrant health volunteer programmes by supporting them in terms of capacity building and social benefits.

*“We should provide some benefits to MHVs which is not only about allowances. According to their volunteerism, they offer their time and opportunities to get paid jobs in these roles. We should provide benefits for them to promote retention, leading to adequate number of MHVs”.*

(C3)

## 4. Discussion

Overall, this study found that health risk communication for migrants during the COVID-19 pandemic was mostly focused on communication activities. Some challenges still existed, including a lack of an overarching body with a clear responsibility for health risk communication for migrants and lack of monitoring and evaluation in the health risk communication process. The health risk communication networks in migrant communities, especially in rural areas, were dependent on a few communication actors, and migrants were hardly able to access health risk information. Another key challenge was that some migrants are not able to access, understand, and obtain relevant and timely information due to different languages used, low literacy, and inadequate access to communication resources. This study emphasises the importance of health risk communication for public health threats including self-care, access to screening test and treatment, and access to information about public health and social measures to protect their rights and well-being.

According to the theoretical implications derived from conceptual frameworks, risk communication cycles can be applied to health risk communication for migrants. It is a continuous loop of health risk communication to identify migrants’ concerns and perceptions, assessing risks to guide communication activities, followed by evaluation and learning, which feeds into improving implementation. By using this framework, the structural gaps are pointed out in addition to assessment of stakeholder coordination and supporting resources to ensure effective health risk communication. The SMCR model is also a useful tool to identify senders and receivers and their relationships through the messages and channels they use to communicate. It also confirms the importance of these four components in complementing the communication process.

Health risk communication in Thailand, including for migrants, is addressed in the plan of the DDC, MOPH. The plan starts by appointing a working committee and stakeholders inside and outside the country; evaluating target groups in terms of number, nationality, and behaviour; disseminating it through national and local public relation organisations, as well as international media; and providing translators, supporting activities in the local area, and evaluation [6]. Nevertheless, this plan is not implemented because there is no national governing body to enforce action. Other countries also faced problems in creating a governmental national plan for health risk communication for migrants. Only a few governments from countries in Europe could accomplish this plan, and to do so they received assistance from NGOs [22]. For instance, Doctors of the World UK, together with the British Red Cross, have produced comprehensive guidance for migrant communities [23], and the IOM published COVID-19 advice for migrant communities in Italy, which was then translated into various languages [24]. This study highlights Thailand’s absence of a nationally created communication body and urges the commitment of the government to reach migrant populations in COVID-19 communication strategies [22]. Therefore, the national body and plan for migrant health, led by public authorities, should develop and implement health risk communication among migrant communities during public health emergencies.

Given the limited dissemination of COVID-19 information in the health sector, another key challenge is how to engage stakeholders from non-health sectors for the planning and implementation of health risk communication strategies in a constructive way. Results showed that migrants search not only for information about the disease and preventive practices, they also search for other information such as access to healthcare and labour or border control measures which are under the responsibility of other ministries. This situation is also reflected in other countries, where most of the information is about preventive measures, while information on COVID-19 testing procedures and how to access healthcare during the pandemic is rarely available [22]. According to practical guidance for risk communication and community engagement (RCCE) for refugees, internally displaced persons (IDPs), migrants, and host communities particularly vulnerable to the COVID-19 pandemic by the WHO and partners, the collaboration and coordination with involved stakeholders such as the government and civil society organisations in health and non-health sectors, community and religious leaders, and influencers can amplify communication impact, avoid duplication, and expand communication targets reached [25]. Singapore applied RCCE by shifting to a bottom-up strategy, for example, the involvement of community leaders and volunteers into policy decision-making and communication activities [26]. Therefore, stakeholder engagement is recommended at all levels to share information, resources, and authorities for health risk communication, and the local level should be empowered as a part of the policy decision-making process.

Key communication processes include monitoring people’s concerns and popular communication channels in target groups by applying a data-driven approach to guide communication objectives, channels, and messages, then evaluating the communication methods [5,6]. In Thailand, communication activities are the main activity, while monitoring and evaluation systems are weakly implemented. The COVID-19 crisis is different from numerous prior health crises due to its dynamic nature, so many monitoring tools should be exercised [27]. For instance, the UNHCR in Spain launched a COVID-19 online questionnaire of 750 refugees and asylum-seekers to identify their concerns during the COVID-19 crisis, and UNHCR in Germany has conducted two virtual focus group discussions to obtain information from target groups about their current situation and needs [27]. In the Thai context, information from the migrant helpline could be analysed, and social platforms such as a MOPH Facebook page could segment migrant users to identify migrants’ perceptions and concerns. Thus, data to understand migrants’ perception in public health emergencies is important to guide more relevant communication strategies and evaluation processes [28].

For communication actors at the local level, social network analysis also provides a likely useful insight. The closeness-centrality is low among migrant workers. This implies difficulty in access to information and emphasises the challenge to improve health risk communication among this group. If the government plans to disseminate the information to a wide range of players, it should work closely with the nodes that contain high betweenness-centrality values [29]. In this case, nodes with high betweenness-centrality were MHVs, PPHO, DPHO, HPH, and LG, and should be targeted as key sources of information in migrant communities. Moreover, the density and frequency of communication flows in urban areas is higher than in rural areas where the information sources are more centralised, and the communication or network tend to rely on limited sources of information. Accordingly, it is necessary to increase the number of centrality nodes, especially in rural areas to decrease dependency to sources of information and ensure easier access to information, strengthening information flows through key communication actors in migrant communities.

The proximity of MHVs to migrants means they play an active role in communication. Therefore, mechanisms to support the function of MHVs should be in place. The literature also mentioned the system challenges for MHVs in terms of incentives and capacity building [30]. There are suggestions to provide financial and non-financial incentives for MHVs such as supporting allowances, benefits, or other recognition of their work. Furthermore, the training courses should be standardised to create a cohort of cultural mediators who bridge the gap between health personnel and migrants [30]. This should be updated for further public health emergency situations, and the mode of training could be complementary face-to-face and online methods. In other countries, volunteers also play important roles in migrant communities through providing peer-to-peer support, developing online activities, and distributing materials [27]. For example, in Romania, they disseminated targeted messaging campaigns for urban refugees and asylum-seekers to raise awareness of the risks of sexual- and gender-based violence in the context of COVID-19 and the services available to survivors provided by volunteers [27]. Therefore, there are opportunities to improve the MHV programme because MHVs are important for migrant health, even outside of health crises. In-person approaches are necessary for those who cannot access the Internet or have low literacy [1].

Another communication channel is print and online media that should be translated into migrants’ languages and promoted through infographics or videos to ensure accessibility and understanding in a timely manner. Findings from this study showed that printed and online media, especially from local actors, are the closest channels to migrants. Channels are presented in Burmese and are relevant to the local context so are easily accessed by migrants. It can be explained from previous literature that migrant workers in these study sites mostly graduated from primary school and had Burmese ethnicity [31]. Although some stayed in Thailand for years, they were still not able to read and understand Thai [31]. A rapid review of evidence from the EU showed that many online governmental COVID-19 materials are only available in the official language [22], so it is necessary to encourage governments to provide bilingual communication materials. From researcher observation, bilingual materials were placed in government or NGO websites, while the homepage language was only in Thai or English. It is important for these websites to be fully accessible to migrants [32]. Another study by Kiyohara (2022) showed that migrants in Japan have accessed Facebook more than multilingual websites and prefer visual materials rather than text messages [33]. Therefore, the sociodemographic data of migrants and their communication preference should be considered by policymakers and health practitioners to improve health risk communication strategies.

There are a few limitations to this study. Firstly, almost all participants were from Myanmar. Therefore, their experiences could not be generalised to all migrants in the country. Further studies need to explore migrants with other characteristics and in other contexts. Secondly, owing to the COVID-19 situation, researchers could not carry out all planned fieldwork due to the lockdown policy, so MHWs and hospital staff were trained to be questionnaire interviewers. This might induce selection bias by selecting respondents from their own circle and interviewer bias, owing to the role of health professionals and communicators in communities. Moreover, we might miss some key informants who had difficulty in contacting such as relatives or friends in the communities. Finally, the self-reporting questionnaire might not exactly reflect the communication network in the community compared to observation in the field.

Regarding policy implications, a national body and plan for migrant health communication should be developed and implemented. For example, in Thailand, the Bureau of Risk Communication and Health Behavior Development and migrant-related organisations partners should play an active role to implement health risk communication plans for migrants, integrated into EOC in the same way as the Thai population. Monitoring tools should be developed such as migrant helplines or new methods to guide communication objectives, messages, and channels on the basis of migrants’ needs. This network is supposed to gather migrant-related information and support materials in migrants’ first language through trusted sources of information. Communication campaigns should be evaluated, and feedback should be incorporated into the communication process. To prepare for further public health threats, national authorities should go beyond the health sector to include other public or private sectors that are responsible for migrants. This should happen with mutual collaboration to provide necessary materials, finance, and human resources to implement a health risk communication plan for all migrants in Thailand.

At the local level, the health risk communication process has to be applied in the same way as at the national level, with sufficient financial support and capacity development. Communication should not rely on a few organisations but should be distributed among actors such as health facilities and local governments in order to ensure easy access, and contents must focus more on up-to-date policies in addition to health information. The local engagement should also include community members such as MHVs and employers as a part of local health risk communication management. The uniqueness of health risk communication in Thailand is the introduction of the MHW and MHV programme which is well-structured and well-dispersed among migrant community. MHWs and MHVs are competent in communicating health risks compared with other traditional health personnel. This is an opportunity to improve the MHV programme, which is not only beneficial for this crisis but also for supporting migrant health more generally. Finally, all communication should be inclusive, regardless of different races, ethnicities, language, cultures, and capabilities.

Although the Thai context is unique, the lessons above are still useful in other countries where the health of migrants is of critical public health concern. This is because the issues of health risk communication are a common concern in many settings. This study emphasised the importance of structural supports and health risk communication processes implemented for migrants. In addition, the communication network should identify many more key communication actors who are close to migrant communities in order to address the problems of over reliance on limited information sources.

## 5. Conclusions

Although there has been progress in implementing health risk communication among migrants in Thailand, and in having the intention to cover migrants within the health risk communication plan, some challenges remain. These include the lack of a governing body with supporting resources, lack of monitoring tools and evaluation processes, centralised information, and the inaccessibility of specific migrant groups. Therefore, the key recommendation is to create a governing body led by the government with intersectional collaboration across sectors in order to support structural change and to implement a health risk communication plan. The health risk communication process should take place at all levels, especially monitoring and evaluation, which is supposed to guide relevant and timely communication activities and feed back into the process while considering migrants’ characteristics and concerns. Moreover, the communication network should identify many more key communication actors who are close or familiar to migrant communities in order to address the problems of over reliance on limited information sources. Key communicators such as MHVs and local media should be supported as part of community engagement.

## Figures and Tables

**Table 1:** Characteristics of key informants from interview.

Code	Positions	Organisations	Gender
Representatives from central public health authorities (A)			
A1	Chief of subdivision	Office of International Cooperation, MOPH	Female
A2	Chief of subdivision	Bureau of Risk Communication and Health Behavior Development, MOPH	Female
A3	Director of division	Primary Health Care Division, MOPH	Male
A4	Chief of subdivision	Primary Health Care Division, MOPH	Female
A5	Deputy director of division	Division of Health Economics and Health Security, MOPH	Male
Representatives from local public health authorities (B)			
B1	Chief of subdivision (disease control)	Provincial Public Health Office, Phuket	Female
B2	Public health technical officer	District Public Health Office, Phuket	Female
B3	Public health technical officer	District Public Health Office, Phuket	Male
B4	Public health technical officer	Phuket City Municipality, Phuket	Male
B5	Chief of subdivision (disease control)	Provincial Public Health Office, Ranong	Male
B6	Director	Health Centre, Ranong	Female
B7	Public health technical officer	Provincial Hospital, Samut Sakhon	Male
B8	Chief of subdivision (health service)	Provincial Public Health Office, Samut Sakhon	Male
B9	Chief of subdivision (health insurance)	Provincial Public Health Office, Samut Sakhon	Female
B10	Chief of subdivision (disease control)	Provincial Public Health Office, Samut Sakhon	Male
Representatives from NGOs (C)			
C1	Staff	Raks Thai Foundation	Male
C2	Staff	World Vision Foundation, Phuket	Female
C3	Staff	World Vision Foundation, Ranong	Female
C4	Staff	Raks Thai Foundation, Samut Sakhon	Male
C5	President	Proud Association	Male
Representatives from international organisations and philanthropy (D)			
D1	Deputy director	Relief and Community Health Bureau, Thai Red Cross Society	Male
D2	Director of office (vulnerable groups)	Thai Health Foundation	Female
D3	Staff (vulnerable groups)	Thai Health Foundation	Male
D4	Staff (migrant health)	International Organisation for Migration, Thailand	Female
D5	Staff (migrant health)	International Organisation for Migration, Thailand	Female
D6	Staff (labour migration)	International Organisation for Migration, Thailand	Female
D7	Staff (border and migrant health)	World Health Organisation, Thailand	Female
Representatives from academia (E)			
E1	Assistant professor	Faculty of Tropical Medicine, Mahidol University	Male
E2	Researcher	Faculty of Tropical Medicine, Mahidol University	Male
E3	Associate professor	Faculty of Communication Arts, Chulalongkorn University	Male
Representatives of MHWs and MHVs (F)			
F1	Migrant health worker	Headquarter District, Phuket	Female
F2	Migrant health volunteer	Headquarter District, Phuket	Female
F3	Migrant health worker	Headquarter District, Ranong	Female
F4	Migrant health volunteer	Headquarter District, Ranong	Male
F5	Migrant health worker	Headquarter District, Samut Sakhon	Female
F6	Migrant health volunteer	Headquarter District, Samut Sakhon	Female

**Table 2:** Characteristics of key informants from social network analysis.

Province	Level	Occupation	Organisation/Community	Gender	Age
Ranong	Province/ district/ subdistrict	Public health technical officer	Provincial public health office	Male	52
		Public health technical officer	District public health office	Female	37
		Nurse	Provincial hospital	Male	41
		Nurse	Health centre (Pak Nam subdistrict)	Female	59
		Nurse	Town municipality	Female	50
		Staff	World Vision Foundation	Female	42
		Manager	Fishery Association	Female	36
		Labour technical officer	Provincial labour office	Female	47
		Migrant health worker	Health centre (Pak Nam subdistrict)	Female	32
		Migrant health worker	World Vision Foundation	Male	53
	Community	Business owner (migrant health volunteer)	Chumthong community	Male	50
		Business owner (village health volunteer)	Pak Nam subdistrict	Female	42
		Manager	E&C Frozen Foodsm, Pak Nam subdistrict	Male	52
		Fishery	Chumthong community	Male	36
		Fishery	Chumthong community	Male	49
		Fishery	Chumthong community	Male	27
		Unemployed	Chumthong community	Female	34
		Fishery	Chumthong community	Male	38
		Fishery	Chumthong community	Male	43
		Fishery	Chumthong community	Male	38
		Unemployed	Chumthong community	Female	48
		Unemployed	Chumthong community	Female	36
		Fishery	Chumthong community	Male	44
		Fishery	Chumthong community	Male	44
		Fishery	Chumthong community	Female	36
		Business owner	Chumthong community	Female	36
Samut Sakhon	Province/ district/ subdistrict	Public health technical officer	Provincial public health office	Male	55
		Public health technical officer	District public health office	Male	52
		Public health technical officer	Provincial hospital	Male	30
		Legal officer	Raks Thai Foundation	Male	28
		Nurse	Health centre (Tha Chin subdistrict)	Female	30
		Sanitation technical officer	Subdistrict municipality (Tha Chin subdistrict)	Female	25
		Migrant health worker	Provincial hospital	Female	27
		Migrant health worker	Raks Thai Foundation	Male	45
	Community	Housemaid (village health volunteer)	Tha Chin village	Female	55
		Housemaid (village health volunteer)	Lang San village	Female	42
		Factory worker (migrant health volunteer)	Wat Noi Nang Hong community	Male	35
		Housemaid (migrant health volunteer)	Ban Auea Arthorn Tha Chin community	Female	37
		Community leader	Ban Auea Arthorn Tha Chin community	Male	49
		Community leader	Wat Noi Nang Hong community	Female	45
		Employer	Fishery factory (Ban Auea Arthorn Tha Chin community)	Male	57
		Employer	Construction company (Wat Noi Nang Hong community)	Male	37
		Accommodation manager	Royal Frame Group Co., Ltd., Bangkok (Wat Noi Nang Hong community)	Male	46
		Accommodation manager	Montri accommodation (Ban Auea Arthorn Tha Chin community)	Female	44
		Unemployed	Ban Auea Arthorn Tha Chin community	Male	28
		Factory worker	Okeanos Co., Ltd., Khokkham subdistrict (Ban Auea Arthorn Tha Chin community)	Female	25
		Factory worker	Thai Union Group Co., Ltd., Thasai subdistrict (Ban Auea Arthorn Tha Chin community)	Male	31
		Construction labour	Taweesamut Enginneering Co., Ltd., Mahachai subdistrict (Ban Auea Arthorn Tha Chin community)	Male	33
		Factory worker	Saksawad Marine Co., Ltd., Na Di subdistrict (Ban Auea Arthorn Tha Chin community)	Female	28
		Unemployed	Ban Auea Arthorn Tha Chin community	Female	35
		Factory worker	DOD Biotech, Tha Chin subdistrict (Wat Noi Nang Hong community)	Female	39
		Factory worker	DOD Biotech, Tha Chin subdistrict (Wat Noi Nang Hong community)	Male	47
		Factory worker	Sin Tai Long Co., Ltd., Tha Chin subdistrict (Wat Noi Nang Hong community)	Male	35
		Factory worker	Lookchinpladao Part., Ltd., Bangyaprak subdistrict (Wat Noi Nang Hong community)	Female	37
		Factory worker	DOD Biotech, Tha Chin subdistrict (Wat Noi Nang Hong community)	Female	29
		Unemployed	Wat Noi Nang Hong community	Male	47

Note: Chumthong community, Pakklong subdistrict; Wat Noi Nang Hong community, Lang San village, Tha Chin subdistrict; Ban Auea Arthorn Tha Chin community, Tha Chin village, Tha Chin subdistrict.

## Data Availability

Not applicable.

## Supplementary Materials

The following supporting information can be downloaded at: https://www.mdpi.com/article/10.3390/tropicalmed7100296/s1, Table S1: Social network analysis in Chumthong migrant community, Ranong; Table S2: Social network analysis in Wat Noi Nang Hong migrant community, Samut Sakhon; Table S3: Social network analysis in Ban Auea Arthorn Tha Chin migrant community, Samut Sakhon.
