# Supplementary material for: Risk Communication Distributed among Migrant Workers during the COVID-19 Crisis in Thailand: Analysis on Structural and Networking Gaps
Source: Trop Med Infect Dis. 2022 Oct 12;7(10):296. doi: 10.3390/tropicalmed7100296 (PMC9607165; doi:10.3390/tropicalmed7100296)
Supplement: Supplementary file 1 [file tropicalmed-07-00296-s001.zip › tropicalmed-1939272-supplementary.pdf]

Supplementary file

Table S1 Social network analysis in Chumthong migrant community, Ranong

Node ID	Node name	Age	Gender	Occupation	Organisation/ Community	In-degree	Out-degree	Betweenness-centrality	Closeness-centrality	Average distance to migrant worker
1	HPH Staff	59	F	Nurse	Health Centre (Pak Nam subdistrict)	5	7	44.0	0.0112	2.3
2	DPHO Staff	37	F	Public Health Technical Officer	District Public Health Office	2	15	119.8	0.0222	2.0
3	PPHO Staff	52	M	Public Health Technical Officer	Provincial Public Health Office	14	11	319.7	0.0118	1.6
4	VHV	42	F	Business Owner	Pak Nam subdistrict	5	12	173.3	0.0102	2.0
5	NGO Staff	42	F	Staff	World Vision Foundation	1	5	1.8	0.0106	2.4
6	Employer/Colleague	52	M	Manager	E&C Frozen Foods Co., Ltd., Pak Nam subdistrict	5	8	86.1	0.0114	2.0
7	Hospital Staff	41	M	Staff	Ranong Hospital	2	4	27.0	0.0769	2.2
8	FA Staff	36	F	Manager	Fishery Association	2	2	2.8	0.0064	2.6
9	PLO Staff	47	F	Staff	Provincial Labour Office	0	6	0	0.0097	2.2
10	LG Staff	50	F	Nurse	Town Municipality	0	6	0	0.0070	2.0
11	HPH MHW	32	F	Migrant Health Worker	Health Centre (Pak Nam subdistrict)	3	8	3.1	0.0083	2.3
12	Migrant 1	36	M	Fishery	Chumthong community	0	3	0	0.0094	N/A
13	Migrant 2	49	M	Fishery	Chumthong community	0	4	0	0.0101	N/A
14	Migrant 3	27	M	Fishery	Chumthong community	0	4	0	0.0097	N/A
15	MHV	50	M	Seller	Chumthong community	11	2	93.4	0.0094	1.6
16	NGO MHW	53	M	Migrant Health Worker	World Vision Foundation	5	6	69.3	0.0108	2.1
17	Migrant 4	34	F	Unemployed	Chumthong community	0	3	0	0.0093	N/A
18	Migrant 5	38	M	Fishery	Chumthong community	0	2	0	0.0080	N/A
19	Migrant 6	43	M	Fishery	Chumthong community	0	3	0	0.0079	N/A
20	Migrant 7	38	M	Fishery	Chumthong community	0	2	0	0.0078	N/A

21	Migrant 8	48	F	Unemployed	Chumthong community	0	2	0	0.0088	N/A
22	Migrant 9	36	F	Unemployed	Chumthong community	0	2	0	0.0088	N/A
23	Migrant 10	44	M	Fishery	Chumthong community	0	2	0	0.0079	N/A
24	Migrant 11	44	M	Fishery	Chumthong community	0	3	0	0.0092	N/A
25	Migrant 12	36	F	Fishery	Chumthong community	0	3	0	0.0098	N/A
26	Migrant 13	36	F	Seller	Chumthong community	0	5	0	0.0097	N/A
27	Central MOPH Staff	-	-	-	-	4	0	0	N/A	2.3
28	Government Staff (Other Sectors)	-	-	-	-	5	0	0	N/A	2.5
29	Community Leader	-	-	-	-	4	0	0	N/A	2.6
30	Accommodation Owner	-	-	-	-	2	0	0	N/A	3.0
31	Broadcasting Media (National Media)	-	-	-	-	8	0	0	N/A	2.3
32	Broadcasting Media (Local Media)	-	-	-	-	4	0	0	N/A	2.8
33	Broadcasting Media (Unspecified)	-	-	-	-	1	0	0	N/A	3.2
34	Printed Media (National Media)	-	-	-	-	3	0	0	N/A	2.5
35	Printed Media (Local Media)	-	-	-	-	16	0	0	N/A	1.6
36	Online Media (National Media)	-	-	-	-	5	0	0	N/A	2.3
37	Online Media (Local Media)	-	-	-	-	7	0	0	N/A	2.2
38	Online Media (Unspecified)	-	-	-	-	12	0	0	N/A	1.9

39	International Media	-	-	-	-	1	0	0	N/A	2.8
40	Other Channels	-	-	-	-	3	0	0	N/A	3.0

Note: KIs are node ID 1-26; Female (F); Male (M); Chumthong community, Pak Nam subdistrict

Table S2 Social network analysis in Wat Noi Nang Hong migrant community, Samut Sakhon

Node ID	Node name	Age	Gender	Occupation	Organisation/ Community	In-degree	Out-degree	Betweenness-centrality	Closeness-centrality	Average distance to migrant worker
1	PPHO staff	55	M	Public Health Technical Officer	Provincial Public Health Office	7	15	58.4	0.0098	2.0
2	Employer/ Colleague 2	37	M	Construction Employer	Wat Noi Nang Hong community	7	11	34.3	0.0099	1.5
3	Community Leader2	45	F	Community Leader	Wat Noi Nang Hong community	8	8	17.6	0.0085	2.2
4	VHV 2	42	F	Housemaid	Lang San village	10	9	19.3	0.0075	2.0
5	MHV 2	35	M	Factory Worker	Wat Noi Nang Hong community	10	9	99.5	0.0110	1.3
6	Hospital MHW	27	F	Migrant Health Worker	Samut Sakhon Hospital	7	5	14.7	0.0067	1.5
7	Hospital Staff	30	M	Public Health Technical Officer	Samut Sakhon Hospital	7	9	15.7	0.0099	1.5
8	DPHO Staff	52	M	Public Health Technical Officer	District Public Health Office	3	3	21.6	0.1000	2.5
9	NGO Staff	28	M	Legal Officer	Raks Thai Foundation	2	6	49.1	0.0094	2.0
10	HPH Staff	30	F	Nurse	Health Centre (Tar Chin subdistrict)	7	9	59.2	0.0101	1.5
11	NGO MHW	45	M	Migrant Health Worker	Raks Thai Foundation	3	11	7.8	0.0086	2.2
12	LG Staff	25	F	Sanitation Technical Officer	Subdistrict Municipality (Tar Chin subdistrict)	3	13	32.4	0.0152	2.0
13	Accommodation Owner 2	46	M	Accommodation Manager	Wat Noi Nang Hong community	5	10	3.7	0.0079	1.8
14	Migrant 1	39	F	Fishery	Wat Noi Nang Hong community	0	9	0.0	0.0086	N/A

15	Migrant 2	37	F	Factory worker	Wat Noi Nang Hong community	0	6	0.0	0.0078	N/A
16	Migrant 3	47	M	Factory worker	Wat Noi Nang Hong community	0	4	0.0	0.0056	N/A
17	Migrant 4	35	F	Factory worker	Wat Noi Nang Hong community	1	5	12.7	0.0060	N/A
18	Migrant 5	29	F	Factory worker	Wat Noi Nang Hong community	1	8	1.5	0.0081	N/A
19	Migrant 6	35	F	Unemployed	Wat Noi Nang Hong community	1	8	11.3	0.0075	N/A
20	Central MOPH Staff	-	-	-	-	4	0	0	N/A	2.5
21	Government Staff (Other Sectors)	-	-	-	-	3	0	0	N/A	3.0
22	Broadcasting Media (National Media)	-	-	-	-	12	0	0	N/A	1.5
23	Broadcasting Media (Local Media)	-	-	-	-	11	0	0	N/A	1.3
24	Broadcasting Media (Unspecified)	-	-	-	-	1	0	0	N/A	3.5
25	Printed Media (National Media)	-	-	-	-	4	0	0	N/A	2.5
26	Printed Media (Local Media)	-	-	-	-	13	0	0	N/A	1.0
27	Printed Media (Unspecified)	-	-	-	-	1	0	0	N/A	3.0
28	Online Media (National Media)	-	-	-	-	8	0	0	N/A	2.2
29	Online Media (Local Media)	-	-	-	-	12	0	0	N/A	1.2

30	Online Media (Unspecified)	-	-	-	-	3	0	0	N/A	2.5
31	International Media	-	-	-	-	2	0	0	N/A	2.2
32	Other Channels	-	-	-	-	2	0	0	N/A	2.5

Note: KIs are node ID 1-19; Female (F); Male (M); Wat Noi Nang Hong community, Lang San village, Tha Chin subdistrict

Table S3 Social network analysis in Ban Auea Arthorn Tha Chin migrant community, Samut Sakhon

Node ID	Node name	Age	Gender	Occupation	Organisation/ Community	In-degree	Out-degree	Betweenness-centrality	Closeness-centrality	Average distance to migrant worker
1	PPHO staff	55	M	Public Health Technical Officer	Provincial Public Health Office	5	13	41.1	0.0094	2
2	Accommodation Owner 1	44	F	Accommodation Manager	Ban Auea Arthorn Tha Chin community	6	12	31.8	0.0094	1.7
3	Employer/ Colleague 1	57	M	Factory Employer	Ban Auea Arthorn Tha Chin community	10	6	22	0.0082	1.3
4	Community Leader 1	49	M	Community leader	Ban Auea Arthorn Tha Chin community	9	10	49.5	0.0093	1.8
5	VHV 1	55	M	Housemaid	Tha Chin village	10	7	75.8	0.0081	2
6	MHV 1	37	F	Housemaid	Ban Auea Arthorn Tha Chin community	8	6	36	0.0072	1.7
7	Hospital MHW	27	F	Migrant Health Worker	Samut Sakhon Hospital	7	5	10.2	0.0067	1.5
8	Hospital Staff	30	M	Public Health Technical Officer	Samut Sakhon Hospital	8	9	50.2	0.0083	1.3
9	DPHO Staff	52	M	Public Health Technical Officer	District Public Health Office	3	3	22.6	0.1000	2.7
10	NGO Staff	28	M	Legal Officer	Raks Thai Foundation	1	7	0	0.0103	2
11	HPH Staff	30	F	Nurse	Health Centre (Tar Chin subdistrict)	4	10	23.9	0.0088	2.2
12	NGO MHW	45	M	Migrant Health Worker	Raks Thai Foundation	3	10	26.3	0.0090	2.2
13	LG Staff	25	F	Sanitation Technical Officer	Subdistrict Municipality (Tar Chin subdistrict)	4	12	102.4	0.0143	2
14	Migrant 1	28	F	Factory Worker	Ban Auea Arthorn Tha Chin community	0	5	0	0.0057	N/A

15	Migrant 2	28	M	Unemployed	Ban Auea Arthorn Tha Chin community	0	5	0	0.0064	N/A
16	Migrant 3	31	M	Fishery	Ban Auea Arthorn Tha Chin community	0	7	0	0.0078	N/A
17	Migrant 4	25	F	Factory worker	Ban Auea Arthorn Tha Chin community	0	7	0	0.0074	N/A
18	Migrant 5	33	M	Construction Worker	Ban Auea Arthorn Tha Chin community	0	6	0	0.0060	N/A
19	Migrant 6	37	M	Factory worker	Ban Auea Arthorn Tha Chin community	1	4	0	0.0048	N/A
20	Central MOPH Staff	-	-	-	-	4	0	0	N/A	2.3
21	Government Staff (Other Sectors)	-	-	-	-	3	0	0	N/A	2.8
22	Family/Friends in Myanmar	-	-	-	-	1	0	0	N/A	2.7
23	Broadcasting Media (National Media)	-	-	-	-	10	0	0	N/A	1.5
24	Broadcasting Media (Local Media)	-	-	-	-	6	0	0	N/A	1.8
25	Broadcasting Media (Unspecified)	-	-	-	-	1	0	0	N/A	3.7
26	Printed Media (National Media)	-	-	-	-	5	0	0	N/A	2
27	Printed Media (Local Media)	-	-	-	-	10	0	0	N/A	1.2
28	Printed Media (Unspecified)	-	-	-	-	1	0	0	N/A	3
29	Online Media (National Media)	-	-	-	-	5	0	0	N/A	2.3

30	Online Media (Local Media)	-	-	-	-	12	0	0	N/A	1.3
31	Online Media (Unspecified)	-	-	-	-	4	0	0	N/A	2
32	International Media	-	-	-	-	1	0	0	N/A	2.7
33	Other channels	-	-	-	-	2	0	0	N/A	2.8

Note: KIs are node ID 1-19; Female (F); Male (M); Ban Auea Arthorn Tha Chin community, Tha Chin village, Tha Chin subdistrict
